# Supplementary material for: Students distracted by electronic devices perform at the same level as those who are focused on the lecture
Source: PeerJ. 2014 Sep 16;2:e572. doi: 10.7717/peerj.572 (PMC4179383; doi:10.7717/peerj.572)
Supplement: Supplemental Information 1 — Main data. [file peerj-02-572-s001.pdf]

Upgrade romesh31

Home My Surveys Survey Services Plans & Pricing + Create Survey

You have a **BASIC account** | To remove the limits of a BASIC account and get unlimited questions, **upgrade now!**

Special Needs Dentistry Lecture

Customer Feedback

Design Survey Collect Responses Analyze Results

View Summary
Browse Responses
Filter Responses
Crosstab Responses
Download Responses
Share Responses

Introducing New Analyze *BETA*
Better charts, easier tools, faster decisions.
Try It Now Learn More

Default Report + Add Report

Response Summary

Total Started Survey: 27
Total Finished Survey: 27 (100%)

PAGE: 1

1. Are you male or female? Create Chart Download

	Response Percent	Response Count
**Male**	33.3%	9
**Female**	**66.7%**	**18**
	**answered question**	**27**
	**skipped question**	**0**

2. I attended this lecture in the following way: Create Chart Download

	Response Percent	Response Count
**In-person at HSDM**	**100.0%**	**27**
**Online through computers at HSDM or HMS**	3.7%	1
**Online through my desktop**	0.0%	0
**Online through my laptop**	3.7%	1
**Online through my iPad**	0.0%	0
**Online using another device not listed here**	0.0%	0
**I did not attend the lecture**	0.0%	0
	**answered question**	**27**
	**skipped question**	**0**

3. Please select the 5 CORRECT STATEMENTS about special needs dentistry and individuals with special health care needs (ISHCN) from the list below:

Create Chart Download

	Response Percent	Response Count
**90% of patients with special needs are not special needs at all**	0.0%	0
**90% of patients with special needs can receive routine dental care like the rest of the population**	66.7%	18
**90% of patients with special needs have an undiagnosed dental problem**	29.6%	8
**90% of patients with special needs have never been to a general dentist**	7.4%	2
**Close to 90% of dental schools do not fulfill accreditation requirements in special needs dentistry**	18.5%	5
**Close to 90% of dental schools do not teach special needs dentistry**	22.2%	6
**Close to 90% of dental school Deans do not believe teaching special needs dentistry is important**	14.8%	4
**Close to 90% of dental schools evaluate special needs dentistry with written tests**	40.7%	11
**Most ISHCN have not used their insurance in the most efficient way**	11.1%	3
**Most ISHCN have the finances but have not purchased insurance**	0.0%	0
**Most ISHCN have better insurance than the general population**	**81.5%**	**22**
**Most ISHCN have a medical issue that makes them ineligible for insurance**	11.1%	3
**There are more ISHCN in the United States than their are uninsured individuals**	37.0%	10
**There are more uninsured individuals in the United States than their are ISHCN**	33.3%	9
**There are more ISHCN in the United States than any other country**	37.0%	10
**There are more ISHCN in Massachusetts than any other state in the United States**	3.7%	1
**Medicaid covers individuals with certain disabilities for dental care**	55.6%	15
**Medicaid covers ISHCN for everything except dental care**	7.4%	2
**In Massachusetts, ISHCN are eligible for Medicaid (MassHealth) but not in any other state**	11.1%	3
	**answered question**	**27**

skipped question	0

4. One important challenge for an ISHCN to access dental care that was mentioned in the talk is

Download

	Response Count
Show Responses	25
answered question	25
skipped question	2

5. During this lecture I checked my email

Create Chart Download

	Response Percent	Response Count
**True**	**59.3%**	**16**
**False**	40.7%	11
If TRUE, please let us know what device you used Show Responses		14
**answered question**		**27**
**skipped question**		**0**

6. During this lecture I sent an email

Create Chart Download

	Response Percent	Response Count
**True**	11.1%	3
**False**	**88.9%**	**24**
If TRUE, please let us know if the email was related to this lecture AND if the recipient is in this lecture with you Show Responses		2
**answered question**		**27**
**skipped question**		**0**

7. During this lecture I checked my Facebook account

Create Chart Download

Response Response

	Response Percent	Response Count
**True**	14.8%	4
**False**	**85.2%**	**23**
	**answered question**	**27**
	**skipped question**	**0**

8. During this lecture I sent a Text message

Create Chart Download

	Response Percent	Response Count
**True**	7.7%	2
**False**	**92.3%**	**24**
If TRUE, please let us know if the text was related to this lecture AND if the recipient is in the lecture with you	Show Responses	1
	**answered question**	**26**
	**skipped question**	**1**

9. I learned the important points about the perceived myths in issues related to special needs and the challenges in access to dental care.

Create Chart Download

	Response Percent	Response Count
**True**	**100.0%**	**26**
**False**	0.0%	0
	**answered question**	**26**
	**skipped question**	**1**

10. What is one thing you would like changed/improved about this lecture?

Download

		Response Count
	**Show Responses**	21
	**answered question**	**21**
	**skipped question**	**6**

Follow Us: Facebook • Twitter • LinkedIn • Our Blog • Google+ • YouTube

Help: FAQs & Tutorials • Contact Support

About Us: Management Team • Board of Directors • Partners • Newsroom • Contact Us • We're Hiring • Sitemap

Policies: Terms of Use • Privacy Policy • Anti-Spam Policy • Security Statement • Email Opt-Out

Dansk • Deutsch • English • Español • Français • 한국어 • Italiano • Nederlands • 日本語 • Norsk • Português • Русский • Suomi • Svenska • 中文(繁體)

Copyright © 1999-2013 SurveyMonkey
